# New Approach for Detection of Normal Alternative Splicing Events and Aberrant Spliceogenic Transcripts with Long-Range PCR and Deep RNA Sequencing

**DOI:** 10.3390/biology10080706

**Published:** 2021-07-23

**Authors:** Vita Šetrajčič Dragoš, Vida Stegel, Ana Blatnik, Gašper Klančar, Mateja Krajc, Srdjan Novaković

**Affiliations:** 1 Department of Molecular Diagnostics, Institute of Oncology Ljubljana, SI-1000 Ljubljana, Slovenia; vsetrajcic@onko-i.si (V.Š.D.); vstegel@onko-i.si (V.S.); gklancar@onko-i.si (G.K.); 2 Biotechnical Faculty, University of Ljubljana, SI-1000 Ljubljana, Slovenia; 3 Cancer Genetics Clinic, Institute of Oncology Ljubljana, SI-1000 Ljubljana, Slovenia; ablatnik@onko-i.si (A.B.); mkrajc@onko-i.si (M.K.); 4 Faculty of Medicine, University of Ljubljana, SI-1000 Ljubljana, Slovenia

**Keywords:** splicing, RNA sequencing, alternative splicing, germline variant, spliceogenic variant, alternative transcript, splicing variant, DNA variant

## Abstract

**Simple Summary:**

RNA splicing defects, caused by genetic variants, are a common molecular mechanism of disease. To detect variants that cause splicing impairment, mRNA-based studies must be performed. Classical mRNA assays are time-consuming, which is why we have validated a new reliable straightforward approach to detect normal alternative splicing events and also splicing aberrations. Using our approach, we were able to reclassify three variants of uncertain significance in NBN and STK11 genes, which is of great importance for a proper clinical management of the patients.

**Abstract:**

RNA sequencing is a promising technique for detecting normal and aberrant RNA isoforms. Here, we present a new single-gene, straightforward 1-day hands-on protocol for detection of splicing alterations with deep RNA sequencing from blood. We have validated our method’s accuracy by detecting previously published normal splicing isoforms of *STK11* gene. Additionally, the same technique was used to provide the first comprehensive catalogue of naturally occurring alternative splicing events of the *NBN* gene in blood. Furthermore, we demonstrate that our approach can be used for detection of splicing impairment caused by genetic variants. Therefore, we were able to reclassify three variants of uncertain significance: *NBN*:c.584G>A, *STK11*:c.863-5_863-3delCTC and *STK11*:c.615G>A. Due to the simplicity of our approach, it can be incorporated into any molecular diagnostics laboratory for determination of variant’s impact on splicing.

## 1. Introduction

Alternative splicing is a process in which a single gene’s pre-mRNA undergoes processing into multiple mature mRNA isoforms. Nearly all human multi-exon genes are involved in alternative splicing. For instance, *BRCA1* gene contains 23 exons, but 63 alternative splicing events are produced by wild-type allele [1]. Understanding the naturally occurring alternative splicing isoforms of clinically relevant genes is of great importance for correct interpretation of splicing assays. RNA splicing defects are a common molecular mechanism of disease, and there are studies demonstrating that RNA sequencing (RNAseq) considerably improves diagnostics yield [2,3,4]. In the study by Yamada et al., the authors showed that the detection rate of deleterious variants increased by 19% if combination of exome and transcriptome analysis was performed, compared with exome sequencing alone [2]. Similarly, Karam et al. reported that DNA sequencing (DNAseq) in combination with RNAseq improved clinical management of 1 in 43 patients in hereditary cancer syndromes [4]. Therefore, identification of splicing defects is of high importance to improve patient’s management. Unfortunately, conventional RNA-based functional assays, such as minigene splicing assays, direct Sanger sequencing and capillary electrophoresis are labor intensive, difficult to interpret and often inconclusive [5]. An additional drawback of the above-mentioned conventional RNA assays is that the maximal fragment’s length suitable for analysis is limited to approximately 1000bp, which makes it impossible to investigate variants detected in long exons such as exon 10 in *BRCA1* and exon 11 in *BRCA2* gene. Therefore, to capture a variant’s complete impact on splicing, whole gene RNA sequencing should be performed. With the development of next generation sequencing, targeted RNAseq or even whole transcriptome sequencing is nowadays technically feasible. However, for small diagnostic laboratories, whole transcriptome sequencing can be financially demanding, and it requires high computational power and storage capacity for RNAseq data analysis [6,7].

Naturally occurring alternative splicing events must be determined by analyzing control samples alongside the patient sample to eliminate the possibility of misinterpreting the variant under investigation as spliceogenic. Previous studies have systematically determined alternative splicing events of genes associated with hereditary breast and/or ovarian cancer, such as *BRCA1, BRCA2, PALB2* and *STK11* [1,8,9]. However, genes associated with rare syndromes or genes with lower penetrance, e.g., *NBN*, remain less studied. Therefore, for small diagnostics laboratories dealing with large numbers of unclassified variants, it is crucial to develop a quick, easy, non-laborious and bioinformatically uncomplicated test for detecting splicing defects to minimize the number of variants of uncertain significance (VUS).

Consequently, the main purpose of our article is to describe a simple method for detecting variants that have an impact on splicing. Along with this, for the first time using our method, a list of alternative splicing events of the *NBN* gene is provided.

## 2. Materials and Methods

### 2.1. Patient Samples

A total of 5 patient blood samples and 6 unrelated control blood samples were collected into Tempus Blood RNA Tube (ThermoFisher, Waltham, MA, USA). Patient samples were carriers of the spliceogenic variants *NF1*:c.122A>T, *NF1*:c.7395-17T>G, *NBN*:c.584G>A, *STK11*:c.863-5_863-3delCTC and *STK11*:c.615G>A. Control samples were used for detection of alternative splicing events in *STK11* and *NBN* genes. Total RNA was isolated from whole blood using Tempus™ Spin RNA Isolation Kit (ThermoFisher).

The present study was approved by the Institutional Review Board of the Institute of Oncology Ljubljana (permission no. OIRIEK00937) and by the National Medical Ethics Committee of Republic of Slovenia (permission no. 0120-339/2019/5). Individual patient consent was waived for this study, as it was a retrospective study, the research involved no risk to the subjects, and the institutional informed consent forms for treatment included consent for the use of patient’s data, materials and/or test results for research purposes. All procedures followed in the present study were therefore in accordance with the ethical standards of the responsible committees on human experimentation (institutional and national) and the Helsinki Declaration of 1975, as revised in 2013.

### 2.2. DNA Sequencing—DNAseq

DNA sequencing was performed as previously described in Setrajcic Dragos et al., 2019 and Klancar et al., 2020 [10,11]. Control samples harboring only undoubtedly benign variants (described in Supplementary Table S2) in the coding region and ±25 nt of intronic sequence of *STK11* and *NBN* gene were selected for alternative splicing isoform discovery.

### 2.3. RNA Sequencing—RNAseq

cDNA synthesis was performed with SuperScript™ IV VILO™ Master Mix (ThermoFisher) using 100ng of total RNA. Primers for genes *STK11*, *NF1* and *NBN* were designed to flank 5′ and 3′UTR. cDNA was amplified with long-range PCR using LongAmp^®^ Taq 2X Master Mix (New England Biolabs) (primer sequences and PCR conditions are described in the Supplementary Table S2).

PCR products were quantified with Qubit (ThermoFisher). Long-range PCR amplicons were used for further library preparation with Nextera XT according to manufactures’ instructions (Illumina). The library was quantified with LabChip^®^ GX Touch™ Nucleic Acid Analyzer (PerkinElmer). The library was paired-end sequenced (2 × 121 cycles) on NextSeq 550 (Illumina).

Raw data files (bcl) were converted to fastq files using bcl2fastq2 tool. FastQC tool was used to determine the quality of NGS data. STAR aligner 2.7.3a was used for alignment of NGS reads to hg19 genome assembly, with the following settings: --outFilterMultimapNmax 2 --outFilterMismatchNmax 20 --chimSegmentMin 0 [12] Samtools was used to create index bam (bam.bai) file [13]. All splicing events were obtained from OutSJ.tab file, produced by STAR. Sashimi plots were created using rmats2sashimi tool. Bioinformatic tools for splicing prediction NNSplice, MaxEntScan, Gene splicer, SpliceSiteFinder-like (included in Alamut visual software) and SpliceAI were used [14].

### 2.4. Alternative Splicing Events Threshold

Junctions covered with a minimum of 20 reads and present in at least two samples or previously published were considered as real splicing junctions. Junctions below the set threshold were regarded as sequencing artifacts and/or biological outliers.

### 2.5. Identification of Splicing Aberrations Caused by Genetic Variant

Genetic variants and alternative splicing events are described following HGVS nomenclature v19.01, where c.1 and r.1 are the A of the ATG translation initiation codon. Reference transcripts NM_000455.4, NM_002485.4 and NM_000267.3 for genes *STK11*, *NBN* and *NF1* were used, respectively. Alternative splicing isoform was defined as any splice junction not defined in the above-mentioned reference transcripts. Splicing isoforms are described using symbols: Δ ((partial) exon skipping), ▼ (intron insertion), p (acceptor shift) and q (donor shift). If multiple cryptic exon inclusion events occurred within the same intron, subsequent letters were added to the event. For example, if three cryptic exon inclusion evets occurred between exons 4 and 5, we described it as ▼4A, ▼4B, ▼4C.

## 3. Results

**Figure 1:**
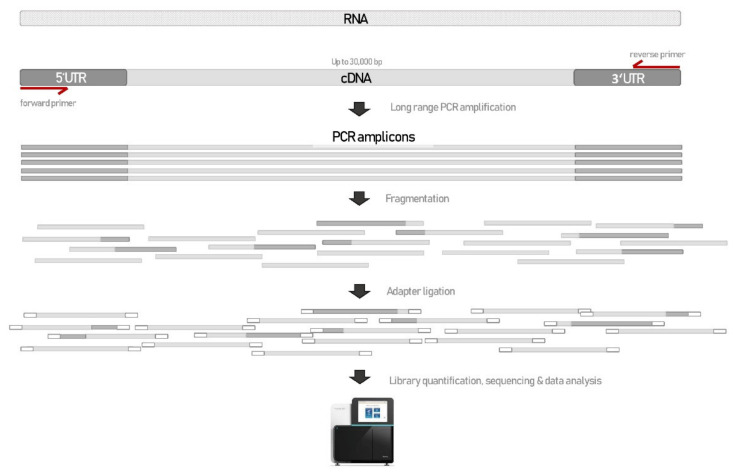
Schematic representation of novel approach used to detect splicing aberration with deep RNAseq.

Here, we present a straightforward 1-day hands-on protocol for detection of splicing alterations in blood with deep RNAseq (cDNA seq), regardless of the gene in question. The test is based on long-range PCR with primers aligning to the 5′UTR and 3′UTR regions of the targeted cDNA. The long PCR amplicon is then fragmented with Nextera transposome and tagged with a universal overhang. Next generation sequencing (NGS) library is further prepared with Illumina’s Nextera XT. Schematic representation of the novel RNAseq method is presented in Figure 1.

### 3.1. Method Confirmation—Alternative Splicing Events in STK11 Gene

**Figure 2:**
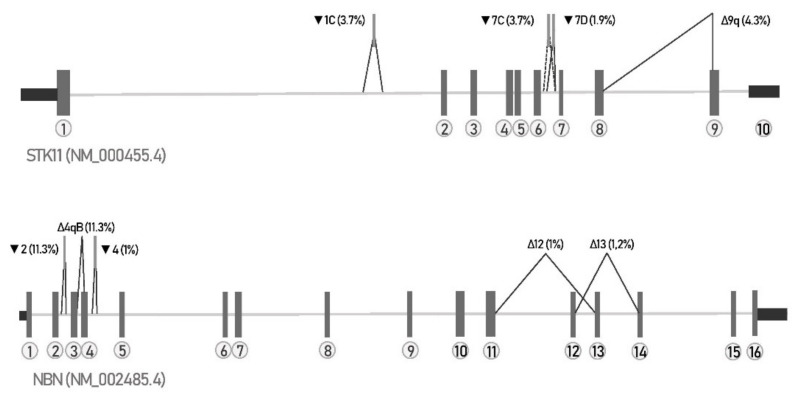
Schematic representation of major *STK11* and *NBN* alternative splicing events. Only splicing events with expression higher than 1% are shown in the image.

The first step in our study was to confirm that our new NGS library preparation and bioinformatics pipeline can reliably detect all major splicing events including exonic and intronic splice-site shift, cryptic exon inclusion and (multiple) exon skipping. Therefore, we decided to determine all naturally occurring splicing junctions of *STK11* gene expressed in blood and compare our results with those previously identified by Brandão et al., 2019. In order to detect even less expressed events, we were aiming for coverage of canonical splice junctions above 100,000×. We were able to detect 36/38 (95%) of previously reported *STK11* splicing junctions, missing one exon skipping and one multi exon skipping event. However, not all previously reported junctions reached our threshold (covered with at least 20 reads and present in at least 2 samples): one junction Δ4–5 was expressed extremely weakly in all 6 samples with the average of 6 reads, whereas junctions ▼1H, ▼1I and ▼7q were expressed in one sample only, but with a considerable number of reads spanning the junction: 116, 175 and 29 reads, respectively. We were unable to detect two previously published junctions of *STK11* gene, Δ2–5 and Δ7. Hence, we aligned our data again to the sequence of the two known events and visually inspected the alignment. No reads mapped to those two events, suggesting they were indeed not present in our data. The splicing event Δ7 was however detected in a patient sample, which harbored a leaky splice-site variant *STK11*:c.863-5_863-3delCTC in intron 6, causing the exon 7 skipping. Four splicing events were predominantly expressed (with a percentage of reads >1%): ▼1C, ▼7C, ▼7D and Δ9q, graphically presented in Figure 2. All four events are predicted to create frameshifts and a premature stop codon, resulting in nonsense-mediated decay or nonfunctional protein. The highest expressed splicing event that can possibly retain protein function was Δ2–3 (0.37%), causing an in-frame deletion of amino acids 98-155. In addition, we detected 18 splicing events that have not been published before (Table 1), suggesting that our approach can be useful for detection of splicing events in *STK11* gene.

### 3.2. Catalogue of Naturally Occurring Splicing Events in NBN Gene

Once the new method was established, we examined the alternative splicing events in *NBN* gene, with an identical approach (Table 2). In the previous studies, 10 alternatively spliced isoforms have been identified with RT-PCR [15,16,17]. Using our approach, we were able to identify all 10 previously described alternative splicing events as well as 49 previously undescribed alternative splicing events. To our knowledge, this is the most extensive catalogue of naturally occurring alternative splicing events of the *NBN* gene. Altogether, we detected 59 alternative splicing events; 11 (Δ2q, Δ3–4, Δ4–5, Δ6–7, Δ12, Δ13qA, Δ13, Δ12–13, Δ14, Δ13–14, Δ12–14) were predicted to be in-frame deletions that can possibly rescue the protein function. In-frame deletions of exon 13 (Δ13) and exon 12 (Δ12) were expressed the highest, with 1.2% and 1%, respectively. Only exons 8, 10 and 11 were not a subject of exon skipping. In *NBN* gene, we were able to detect two splicing events affecting 3′UTR region, which might actually be alternative 3′UTR isoforms [18].The highest expressed alternative splicing event was cryptic exon inclusion▼2 (11.3%), which corresponds to exon 3 in NCBI reference sequence NM_001024688.2. Events present in more than 1% are depicted schematically in Figure 2. All splicing junctions produced by STAR aligner of both studied genes are listed in the Supplementary Table S1.

### 3.3. Detection of Known Spliceogenic Variants

**Figure 3:**
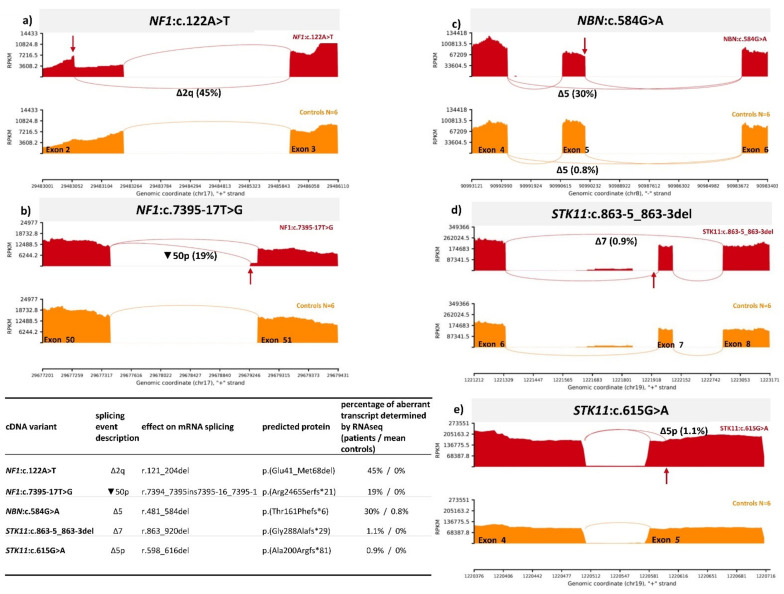
Sashimi plots, representing the splicing impairment caused by variant (**a**) *NF1*:c.122A>T (**b**) *NF1*:c.7395-17T>G (**c**) *NBN*:c.584G>A p.(Ser195Asn) (**d**) *STK11*:c.863-5_863-3delCTC (**e**) *STK11*:c.615G>A p.(Ala205=). Red arrows label variant’s location. Red sashimi plots represent carriers. Orange plots represent control samples (N = 6). Junctions without label are normal transcripts; junctions with labels represent aberrant transcripts.

To verify that our method is capable of detecting an abnormal splicing pattern caused by a spliceogenic variant, we used our method to test two variants previously characterized as spliceogenic. Impact on splicing for both variants has been previously established with direct Sanger sequencing and capillary electrophoresis (CE) [10]. Variant 1 is *NF1*:c.122A>T r.121_204del, which creates an exonic donor shift leading to deletion of 84 bp of exon 2 (Δ2q). Assessed by CE, *NF1*:c.122A>T induced transcript represented 52% in comparison to 48% of full-length transcript. Variant 2 is *NF1*:c.7395-17T>G r.7394_7395ins7395-16_7395-1, which causes an acceptor shift and retention of last 16 bp of intron 50 (▼50p). Aberrant transcript was present in 17.3% determined by CE. Indeed, our new RNAseq protocol successfully identified the disruption of normal splicing and determined the exact splicing junction in both samples (Figure 3). Additionally, according to the new RNAseq method, the fraction of aberrant transcripts caused by *NF1* variants was 45% and 19% for *NF1*:c.122A>T and *NF1*:c.7395-17T>G, respectively.

### 3.4. Determination of Spliceogenicity of VUS

Three variants in *NBN* or *STK11* genes, which were bioinformatically predicted to cause splicing impairment, were selected for the analysis: *NBN*:c.584G>A, *STK11*:c.863-5_863-3delCTC and *STK11*:c.615G>A. The results are visualized in Figure 3.

***NBN*:c.584G>A** is located in the ultimate position of exon 5, which is predicted to completely abolish natural donor splice site. We observed strengthening of out-of-frame exon 5 skipping (Δ5) in the variant carrier. Analyzing the junction data, Δ5 was present in 30% of junctions in *NBN*:c.584G>A carrier in comparison to 0.8% in controls. After inspecting the alignment file at the position c.584, only wild-type nucleotide G was detected, implicating that the *NBN*:c.584G>A variant does not form any full-length transcript.

***STK11*:c.863-5_863-3delCTC** variant is located in intron 6 and is predicted to decrease the strength of native acceptor splice site. RNAseq analysis has revealed an abnormal transcript that lacks exon 7 (Δ7). However, the out-of-frame Δ7 transcript was minorly expressed (only in 0.9%), implying that *STK11*:c.863-5_863-3delCTC variant causes low leaky splicing abnormality. The control samples did not harbor Δ7 transcript.

Similarly, ***STK11*:c.615G>A** creates minor splicing defect (1.1%) by introducing a de novo acceptor splice site. The variant causes minor frameshift deletion of first 19 nucleotides of exon 5 (Δ5p). When inspecting mapped data, mutated A nucleotide at the position c.615 was present in 49% of the reads, which confirms that the splicing abnormality is minor. The Δ5p transcript was not present in the controls.

## 4. Discussion

RNA-based experiments are often performed in diagnostics laboratories in order to identify variants that cause RNA splicing impairment [19,20,21,22]. RT-PCR followed by capillary electrophoresis and Sanger sequencing are golden standards for determining variants spliceogenicity. However, such experiments are limited to the location of the variant, requiring multiple PCR reactions for different variants in the same gene. Any laboratory, no matter how big or small, requires a reliable straightforward method to determine variants’ effect on splicing for precise variant classification. Here, we present a simple RNAseq approach that efficiently detects splicing junctions, which can be implemented by any laboratory with an access to an NGS instrument.

The first aim of our study was to evaluate if our pipeline is sensitive enough to detect previously determined naturally occurring alternative splicing events of *STK11* gene. We were able to detect 95% of all splicing events and furthermore detect 18 previously undetected splicing events. One previously described event Δ7 was not detected by our analysis in the control samples included in the study [9]. Nevertheless, we were able to detect the identical event Δ7 in a patient with a rare leaky splice-site variant located in intron 6 of *STK11* gene (*STK11*:c.863-5_863-3delCTC) demonstrating that the assay and the pipeline are able to detect such a splicing event, but the transcript was not present in our data. An additional previously described transcript that was not present in our dataset was Δ2–5. These two events may be population specific, as they were detected in all four samples in a study by Brandão et al., 2019, but were not seen in a single sample in our study (Slovenian population). Notably, we were able to detect all types of splicing events: exon skipping, multiple exon skipping, donor/acceptor shift, cryptic exon inclusion and mixed splicing events. Once the method was established, we characterized alternative splicing patterns of the *NBN* gene. This is the first time, to our knowledge, that the naturally occurring splicing events of *NBN* gene were characterized in depth. Ten previously identified alternative splicing events were also detected by our approach [15,16,17]. This catalogue of alternatively spliced transcripts (Table 2) is an important asset for further characterization of possible spliceogenic variants. Interestingly, exons 8 (amino acids 299-332), 10 and 11 (amino acids 375-615) were not subjected to alternative exon skipping. It might be that these exons are essential for protein function. Indeed, the nibrin protein was shown to interact with mTOR/Rictor/SIN1 complex at the amino acid residues 221–402 [23].

Crucial for any RNAseq experiment is its ability to accurately detect splicing impairment [24]. To test that our assay can detect abnormal splicing, two well-studied splice altering *NF1* variants were examined. Our assay correctly identified abnormal splicing junctions that arose due to damaging *NF1*:c.122A>T and *NF1*:c.7395-17T>G variants. Moreover, the percentage of aberrant transcript determined by CE and RNAseq was similar for both variants, meaning the novel RNAseq method can reliably quantify the aberrant versus normal transcript. *NF1*:c.7395-17T>G variant produced 19% of aberrant transcript, which might be due to partial degradation of truncated mRNA by nonsense-mediated decay pathway.

Additionally, our method was used to determine splicoegenicity of three variants of uncertain significance. One variant *NBN*:c.584G>A was shown to completely disturb mRNA splicing by inducing out-of-frame exon 5 skipping and was therefore reclassified as likely pathogenic (ACMG/AMP criteria applied: PS3, PM2, PP3). Two variants *STK11*:c.863-5_863-3delCTC and *STK11*:c.615G>A were determined to cause minor leaky splicing, as they were expressed in extremely low fractions. Moreover, the carriers of both variants did not have any clinical characteristics of Peutz–Jeghers syndrome, which is caused by *STK11* pathogenic variants. Both variants were therefore reclassified as likely benign (ACMG/AMP criteria applied: BS3, BP5, PM2).

Here, we show that our assay can indeed detect altered transcripts, both complete splicing aberrations and leaky splicing, and can be therefore used as a complementary test in molecular diagnostics laboratories to characterize variants’ effect on splicing. Our approach might be used as an alternative method to targeted RNA sequencing or whole transcriptome sequencing when examining one gene of interest.

The main advantage of our approach compared with the targeted RNAseq is that there is no need to design enrichment probes. Designing enrichment probes can be challenging especially for poorly researched genes [25,26]. Furthermore, targeted RNAseq assays are frequently designed and validated for a specific gene panel, which makes it difficult to add or remove genes of interest [26]. An additional advantage of our assay in comparison to targeted or transcriptome sequencing is the possibility to study a single gene of interest, achieving a higher coverage crucial for detection of events expressed in lower fractions. Our assay can be customized for nearly any gene of interest, which makes it highly suitable for laboratories dealing with rare genetic syndromes. The method is limited to fresh tissue samples or cell cultures, since the RNA has to be of high quality so that it is amplifiable with long-range PCR. An additional limitation is the size of the cDNA, which needs to be amplifiable with PCR. In this study, the maximal length of cDNA, which was successfully amplified and analyzed, was 12kb (*NF1* gene).

The limitation of our approach in detecting natural events of *NBN* gene was that DNA sequencing of control samples only included exon regions and 25 bp of intronic sequence. Although deep intronic variants were not ruled out, we avoided rare genetic variants by only counting junctions detected in two or more control samples. Additionally, the newly detected splicing events in our study were not confirmed with an alternative method. To further improve our protocol, molecular barcodes could be used in order to partially avoid biased amplification of certain splicing events. An additional drawback of this study is the low number of samples used for the catalogue of natural splicing events. Moreover, the catalogue of naturally occurring splicing isofroms was conducted with short-read sequencing, which may cause mapping errors. The catalogue could be improved by confirming the isoforms with long-read sequencing, such as single-molecule real-time or nanopore sequencing. Importantly, with our approach, we are only able to determine splicing events not the whole full-length splicing isoforms, which can be achieved with long-read sequencing.

## 5. Conclusions

In conclusion, our novel single-gene assay is a fast, straightforward and cost-efficient technique for discovering splicing impairment. All that a laboratory requires is a set of primers that align to the 5′ and 3′UTR region of the gene of interest, long-range PCR amplification, Nextera XT library preparation kit and access to an NGS instrument. RNAseq data can be analyzed on any desktop computer, without the need for high computational power. Low sequencing cost and low computational power for data analysis make the method accessible even to laboratories with limited budgets. The turnaround time starting from RNA isolation to loading a sequencing library onto an NGS instrument is around 10 h (the duration of the experiment depends on the length of cDNA that needs to be amplified with PCR). Our method can be easily adopted in diagnostics laboratories, as the assay can be performed in a time frame necessary for clinical testing. Importantly, when the method is applied in the clinical laboratory, it should always include control samples for excluding normally present splicing events in the diagnostics sample.

## Figures and Tables

**Table 1:** Splicing events of *STK11* gene detected by new RNAseq approach from control samples in comparison with data published by Brandão et al., 2019 [9].

RNA Consequence	Junction Description	Average Number of Reads Supporting the Junction (N = 6)	Mean Percentage of Junction Reads †	Biotype	Percentage of Samples with Observed Junction	Detected by Brandão et al., 2019
r.-272_-186del	Δ5′UTR	64	0.026	terminal modification	33	no
r.-245_-209del	Δ5′UTR	56	0.023	terminal modification	50	no
r.-258_-185del	Δ5′UTR	150	0.061	terminal modification	50	no
r.-323_597del	Δ5′UTR	86	0.029	terminal modification	33	no
r.290_291ins290+2456_290+2554	▼1A	608	0.207	cryptic exon inclusion	67	yes
r.290_291ins290+5106_290+5326	▼1B	44	0.015	cryptic exon inclusion	50	yes
r.290_291ins291-2149_291-2019	▼1C	10,912	3.717	cryptic exon inclusion	100	yes
r.290_291ins291-2038_291-102	▼1D	70	0.024	cryptic exon inclusion	83	yes
r.290_291ins291-2897_291-2755	▼1E	72	0.025	cryptic exon inclusion	33	no
r.290_291ins291-2149_291-1782	▼1F	105	0.071	cryptic exon inclusion	67	no
	▼1G	146	0.050	cryptic exon inclusion	100	yes
r.290_291ins290+114_290+190 ‡	▼1H	116	0.040	cryptic exon inclusion	17	yes
r.290_291ins291-2149_291-1324 ‡	▼1I	175	0.060	cryptic exon inclusion	17	yes
intron 1 junction	/	293,569	/	intron 1 junction	100	yes
r.374delinsAC	Δ2pA	405	0.141	exonic donor shift	100	no
r.374insA_375delG	Δ2pB	117	0.041	exonic donor shift	100	no
r.373_376del	Δ2,3q	274	0.096	exonic acceptor shift	100	no
r.291_464del	Δ2–3	1140	0.373	multiple exon skipping	100	yes
intron 2 junction	/	286,038	/	intron 2 junction	100	yes
r.373_378del	Δ3q	227	0.079	exonic acceptor shift	67	no
intron 3 junction	/	316,895	/	intron 3 junction	100	yes
r.465_597del	Δ4	532	0.186	exon skipping	100	yes
r.490_653del	Δ4p,Δ5q	67	0.022	mixed	50	no
r.465_734del §	Δ4–5	6	0.002	multiple exon skipping	100	yes
r.465_920del	Δ4–7	62	0.023	multiple exon skipping	100	yes
intron 4 junction	/	254,161	/	intron 4 junction	100	yes
r.706_734del	Δ5	342	0.125	exon skipping	83	yes
r.734del	Δ5p	29	0.010	exonic donor shift	83	no
intron 5 junction	/	295,148	/	intron 5 junction	100	yes
r.862del	Δ6p	36	0.015	exonic donor shift	100	no
r.862_863ins862+281_863-103	▼7A	1545	0.661	cryptic exon inclusion	100	yes
r.862_863ins862+286_863-103	▼7B	1218	0.521	cryptic exon inclusion	100	yes
r.862_863ins863-283_863-103	▼7C	3230	1.382	cryptic exon inclusion	100	yes
r.862_863ins863-253_863-103	▼7D	4570	1.956	cryptic exon inclusion	100	yes
r.862_863ins863-195_863-103	▼7E	186	0.079	cryptic exon inclusion	67	no
r.858_862del+r.862_863ins863-125_863-103	▼7F	16	0.007	mixed	33	no
r.862_863ins863-126_863-103	▼7G	25	0.011	cryptic exon inclusion	33	no
r.862_863ins863-125_863-103	▼7H	202	0.087	cryptic exon inclusion	100	no
r.820_921ins921-34_921-1 ‡	▼7q	29	0.012	intronic acceptor shift	17	yes
intron 6 junction	/	233,667	/	intron 6 junction	100	yes
r.920_921ins921-105_921-1	▼8qA	185	0.074	intronic acceptor shift	100	yes
r.920_921ins921-87_921-1	▼8qB	186	0.074	intronic acceptor shift	83	yes
intron 7 junction	/	250,858	/	intron 7 junction	100	yes
r.1180_1181ins1108+466_1108+600	▼8A	749	0.331	cryptic exon inclusion	100	yes
r.1180_1181ins_1180+1_1108+187	▼8p	141	0.062	intronic donor shift	33	no
r.1180_1181ins1108+466_1109-641	▼8B	95	0.042	cryptic exon inclusion	50	yes
intron 8 junction	/	226,042	/	intron 8 junction	100	yes
r.1109_1113del	Δ9q	9881	4.371	exonic acceptor shift	100	yes
r.1109_*16del	Δ9	67	0.047	exon skipping	33	yes
intron 9 junction	/	56,173	/	intron 9 junction	100	yes

†—calculation method that determines the percentage of detected junctions, adapted from Davy et al., 2017; ‡—events detected in one sample only; §—events covered with less than 20 reads.

**Table 2:** Splicing events of *NBN* (NBS1) gene detected by new RNAseq approach from control samples.

RNA Consequence	Junction Description	Average Number of Reads Supporting the Junction (N = 6)	Mean Percentage of Junction Reads †	Biotype	Percentage of Samples with Observed Junction	Detected by Varon et al., 2006
r.37_38ins37+466_37+648	▼1A	72	0.179	cryptic exon inclusion	50	No
r.37_38ins37+698_37+779	▼1B	33	0.080	cryptic exon inclusion	50	No
intron 1 junction	/	52,217	/	intron 1 junction	100	No
r.38_40del	Δ2q	17	0.033	exonic acceptor shift	67	No
r.171_172ins172-479_172-430	▼2	12,105	11.297	cryptic exon inclusion	100	yes
r.171_172ins172-27_172-1	▼3q	50	0.070	intronic acceptor shift	83	No
r.171_172ins171+1_171+4	▼4p	35	0.048	intronic donor shift	100	No
intron 2 junction	/	72,077	/	intron 2 junction	100	No
r.38_171del	Δ2	31	0.169	exon skipping	83	No
intron 3 junction	/	115,981	/	intron 3 junction	100	No
r.172_320del	Δ3–4	21	0.020	multiple exon skipping	33	No
r.172_320del	Δ3	137	0.145	exon skipping	83	No
r.321_325del	Δ4qA	37	0.032	exonic acceptor shift	67	No
r.321_361del	Δ4qB	3341	2.881	exonic acceptor shift	100	No
r.172_361del NM_001024688.2		31	/	exon skipping+exonic acceptor shift	83	No
r.172_361del	Δ3+4qC	118	0.126	exon skipping+exonic acceptor shift	67	No
r.480_481ins480+306_480+395	▼4	1294	1.041	cryptic exon inclusion	100	No
intron 4 junction	/	138,006	/	intron 4 junction	100	No
r.38_480del	Δ2–4	24	0.023	multiple exon skipping	83	No
r.321_480del	Δ4	58	0.045	exon skipping	50	No
r.172_480del	Δ3–4	43	0.040	multiple exon skipping	33	No
r.481del	Δ5qB	22	0.016	exonic acceptor shift	100	No
intron 5 junction	/	135,653	/	intron 5 junction	100	No
r.481_584del	Δ5	891	0.651	exon skipping	100	Yes
r.321_584del	Δ4–5	161	0.116	multiple exon skipping	100	Yes
r.172_584del	Δ3–5	37	0.035	multiple exon skipping	33	No
r.38_584del	Δ2–5	164	0.174	multiple exon skipping	83	No
r.589del	Δ6qB	86	0.064	exonic acceptor shift	100	No
intron 6 junction	/	139,974	/	intron 6 junction	100	No
r.585_702del	Δ6	75	0.054	exon skipping	50	No
r.703_820del	Δ7q	127	0.090	exonic acceptor shift	50	Yes
intron 7 junction	/	179,463	/	intron 7 junction	100	No
r.585_896del	Δ6–7	118	0.075	multiple exon skipping	83	Yes
r.481_896del	Δ5–7	202	0.224	multiple exon skipping	67	No
r.38_896del	Δ2–7	61	0.052	multiple exon skipping	67	No
r.897del	Δ8q	17	0.010	exonic acceptor shift	100	No
r.994_995ins994+1178_995-1769	▼8A	86	0.041	cryptic exon inclusion	67	No
r.994_995ins995-1769_995-1604	▼8B	34	0.015	cryptic exon inclusion	33	No
intron 8 junction	/	202,305	/	intron 8 junction	100	No
r.1124_1125ins1124+703_1124+760	▼9	391	0.220	cryptic exon inclusion	100	Yes
intron 9 junction	/	181,550	/	intron 9 junction	100	No
r.995_1124del	Δ9	173	0.270	exon skipping	100	No
intron 10 junction	/	127,561	/	intron 10 junction	100	No
r.1398del	Δ11qA	29	0.023	exonic acceptor shift	100	No
r.1398_1403del	Δ11qB	83	0.065	exonic acceptor shift	33	No
r.1398_1471del	Δ11qC	70	0.055	exonic acceptor shift	83	No
r.1845_1846ins1845+1521_1845+1597	▼11	22	0.030	cryptic exon inclusion	33	No
r.1845_1846ins1846-23_1846-1	▼12q	68	0.052	intronic acceptor shift	83	No
intron 11 junction	/	132,009	/	intron 11 junction	100	No
r.1846_1849del	Δ12q	46	0.035	exonic acceptor shift	67	No
intron 12 junction	/	128,083	/	intron 12 junction	100	No
r.1896_1914del	Δ12p	152	0.118	exonic donor shift	100	No
r.1846_1914del	Δ12	894	1.030	exon skipping	100	No
r.1915_1932del	Δ13qA	316	0.247	exonic acceptor shift	100	No
r.1915_2009del	Δ13qB	114	0.089	exonic acceptor shift	67	Yes
intron 13 junction	/	128,636	/	intron 13 junction	100	No
r.1915_2070del	Δ13	1576	1.228	exon skipping	100	Yes
r.1846_2070del	Δ12–13	96	0.074	multiple exon skipping	83	No
r.2184_2185ins2184+417_2184+464	▼14A	59	0.042	cryptic exon inclusion	100	No
r.2184_2185ins2184+1511_2184+1578	▼14B	58	0.036	cryptic exon inclusion	67	No
r.2184_2185ins2185-735_2185-610	▼14C	76	0.059	cryptic exon inclusion	83	No
r.2184_2185ins2185-718_2185-610	▼14D	184	0.149	cryptic exon inclusion	100	Yes
r.2184_2185ins2185-4_2185-1ins	▼14q	33	0.026	intronic acceptor shift	33	No
intron 14 junction	/	123,705	/	intron 14 junction	100	No
r.2071_2184del	Δ14	98	0.077	exon skipping	100	No
r.1915_2184del	Δ13–14	45	0.073	multiple exon skipping	83	No
r.1846_2184del	Δ12–14	222	0.174	multiple exon skipping	100	Yes
intron 15 junction	/	136,201	/	intron 15 junction	100	No
r.2185_2234del	Δ15	70	0.054	exon skipping	83	No
r.1915_2234del	Δ13–15	100	0.075	multiple exon skipping	100	No
r.*39_*541del	Δ3′UTR	93	0.068	terminal modification	67	No
r.2003_*1085del	Δ13p+Δ14–15	74	0.058	multiple exon skipping+exonic donor shift	33	No
r.*1076_*1143del	Δ3′UTR	27	0.020	terminal modification	33	No

†—calculation method that determines the percentage of detected junctions, adapted from Davy et al., 2017.

## Data Availability

The data presented in this study are available upon request from the corresponding author. The data are not publicly available due to patients’ privacy.

## Supplementary Materials

The following are available online at https://www.mdpi.com/article/10.3390/biology10080706/s1, Table S1: naturally occurring splicing junctions of *NBN* and *STK11* genes in the blood of six control samples, obtained with RNAseq and STAR aligner; Table S2: primer sequences and PCR protocol used for long-range amplification of *NBN*, *STK11* and *NF1* genes. Benign variants detected in *NBN* and *STK11* genes in six controls with DNAseq.

## References

[1] Colombo M., Blok M.J., Whiley P., Santamariña M., Gutiérrez-Enríquez S., Romero A., Garre P., Becker A., Smith L.D., De Vecchi G. (2014). Comprehensive annotation of splice junctions supports pervasive alternative splicing at the BRCA1 locus: A report from the ENIGMA consortium. Hum. Mol. Genet..

[2] Yamada M., Suzuki H., Shiraishi Y., Kosaki K. (2019). Effectiveness of integrated interpretation of exome and corresponding transcriptome data for detecting splicing variants of genes associated with autosomal recessive disorders. Mol. Genet. Metab. Rep..

[3] Cartegni L., Chew S.L., Krainer A.R. (2002). Listening to silence and understanding nonsense: Exonic mutations that affect splicing. Nat. Rev. Genet..

[4] Karam R., Conner B., LaDuca H., McGoldrick K., Krempely K., Richardson M.E., Zimmermann H., Gutierrez S., Reineke P., Hoang L. (2019). Assessment of Diagnostic Outcomes of RNA Genetic Testing for Hereditary Cancer. JAMA Netw. Open.

[5] Whiley P.J., De La Hoya M., Thomassen M., Becker A., Brandão R., Pedersen I.S., Montagna M., Menéndez M., Quiles F., Gutiérrez-Enríquez S. (2014). Comparison of mRNA splicing assay protocols across multiple laboratories: Recommendations for best practice in standardized clinical testing. Clin. Chem..

[6] Kulkarni P., Frommolt P. (2017). Challenges in the Setup of Large-scale Next-Generation Sequencing Analysis Workflows. Comput. Struct. Biotechnol. J..

[7] Ozsolak F., Milos P.M. (2011). RNA sequencing: Advances, challenges and opportunities. Nat. Rev. Genet..

[8] Davy G., Rousselin A., Goardon N., Castéra L., Harter V., Legros A., Muller E., Fouillet R., Brault B., Smirnova A.S. (2017). Detecting splicing patterns in genes involved in hereditary breast and ovarian cancer. Eur. J. Hum. Genet..

[9] Brandão R.D., Mensaert K., López-Perolio I., Tserpelis D., Xenakis M., Lattimore V., Walker L.C., Kvist A., Vega A., Gutiérrez-Enríquez S. (2019). Targeted RNA-seq successfully identifies normal and pathogenic splicing events in breast/ovarian cancer susceptibility and Lynch syndrome genes. Int. J. Cancer.

[10] Setrajcic Dragos V., Blatnik A., Klancar G., Stegel V., Krajc M., Blatnik O., Novakovic S. (2019). Two novel NF1 pathogenic variants causing the creation of a new splice site in patients with neurofibromatosis type I. Front. Genet..

[11] Klančar G., Blatnik A., Šetrajčič Dragoš V., Vogrič V., Stegel V., Blatnik O., Drev P., Gazič B., Krajc M., Novaković S. (2020). A Novel Germline MLH1 In-Frame Deletion in a Slovenian Lynch Syndrome Family Associated with Uncommon Isolated PMS2 Loss in Tumor Tissue. Genes (Basel).

[12] Dobin A., Gingeras T.R. (2015). Mapping RNA-seq Reads with STAR. Curr. Protoc. Bioinform..

[13] Li H., Handsaker B., Wysoker A., Fennell T., Ruan J., Homer N., Marth G., Abecasis G., Durbin R. (2009). The Sequence Alignment/Map format and SAMtools. Bioinformatics.

[14] Jaganathan K., Kyriazopoulou Panagiotopoulou S., McRae J.F., Darbandi S.F., Knowles D., Li Y.I., Kosmicki J.A., Arbelaez J., Cui W., Schwartz G.B. (2019). Predicting Splicing from Primary Sequence with Deep Learning. Cell.

[15] Varon R., Dutrannoy V., Weikert G., Tanzarella C., Antoccia A., Stöckl L., Spadoni E., Krüger L.-A., di Masi A., Sperling K. (2006). Mild Nijmegen breakage syndrome phenotype due to alternative splicing. Hum. Mol. Genet..

[16] Tessitore A., Biordi L., Flati V., Toniato E., Marchetti P., Ricevuto E., Ficorella C., Scotto L., Giannini G., Frati L. (2003). New mutations and protein variants ofNBS1 are identified in cancer cell lines. Genes Chromosom. Cancer.

[17] Takakuwa T., Luo W.-J., Francisca Ham M., Aozasa K. (2004). A 50-bp insertion from intron 2 between exons 2 and 3 ofNBS1 may be a spliced variant. Genes, Chromosom. Cancer.

[18] Mayr C. (2016). Evolution and Biological Roles of Alternative 3′UTRs. Trends Cell Biol..

[19] Landrith T., Li B., Cass A.A., Conner B.R., LaDuca H., McKenna D.B., Maxwell K.N., Domchek S., Morman N.A., Heinlen C. (2020). Splicing profile by capture RNA-seq identifies pathogenic germline variants in tumor suppressor genes. Npj Precis. Oncol..

[20] Farber-Katz S., Hsuan V., Wu S., Landrith T., Vuong H., Xu D., Li B., Hoo J., Lam S., Nashed S. (2018). Quantitative Analysis of BRCA1 and BRCA2 Germline Splicing Variants Using a Novel RNA-Massively Parallel Sequencing Assay. Front. Oncol..

[21] Casadei S., Gulsuner S., Shirts B.H., Mandell J.B., Kortbawi H.M., Norquist B.S., Swisher E.M., Lee M.K., Goldberg Y., O’Connor R. (2019). Characterization of splice-altering mutations in inherited predisposition to cancer. Proc. Natl. Acad. Sci. USA.

[22] Krivokuca A., Dragos V.S., Stamatovic L., Blatnik A., Boljevic I., Stegel V., Rakobradovic J., Skerl P., Jovandic S., Krajc M. (2018). Novel BRCA1 splice-site mutation in ovarian cancer patients of Slavic origin. Fam. Cancer.

[23] Wang J.-Q., Chen J.-H., Chen Y.-C., Chen M.-Y., Hsieh C.-Y., Teng S.-C., Wu K.-J. (2013). Interaction between NBS1 and the mTOR/Rictor/SIN1 Complex through Specific Domains. PLoS ONE.

[24] Houdayer C., Caux-Moncoutier V., Krieger S., Barrois M., Bonnet F., Bourdon V., Bronner M., Buisson M., Coulet F., Gaildrat P. (2012). Guidelines for splicing analysis in molecular diagnosis derived from a set of 327 combined in silico/in vitro studies on BRCA1 and BRCA2 variants. Hum. Mutat..

[25] Kukurba K.R., Montgomery S.B. (2015). RNA Sequencing and Analysis. Cold Spring Harb. Protoc..

[26] Hrdlickova R., Toloue M., Tian B. (2017). RNA-Seq methods for transcriptome analysis. Wiley Interdiscip. Rev. RNA.

